# Relationship between salt consumption measured by 24-h urine collection and blood pressure in the adult population of Vitória (Brazil)

**DOI:** 10.1590/1414-431X20154455

**Published:** 2015-06-30

**Authors:** S.L. Rodrigues, P.R. Souza, E.B. Pimentel, M.P. Baldo, D.C. Malta, J.G. Mill, C.L. Szwarcwald

**Affiliations:** 1 Departamento de Ciências Fisiológicas, Centro de Ciências da Saúde, Universidade Federal do Espírito Santo, Vitória, ES, Brasil; 2 Centro de Informação Científica e Tecnológica, Fundação Oswaldo Cruz, Rio de Janeiro, RJ, Brasil; 3 Departamento de Vigilância de Doenças e Agravos Não Transmissíveis e Promoção da Saúde, Ministério da Saúde, Brasília, DF, Brasil

**Keywords:** Sodium, Salt intake, Blood pressure, Hypertension, 24-h urine collection, Population-based study

## Abstract

High salt intake is related to an increase in blood pressure and development of hypertension. However, currently, there are no national representative data in Brazil using the gold standard method of 24-h urine collection to measure sodium consumption. This study aimed to determine salt intake based on 24-h urine collection in a sample of 272 adults of both genders and to correlate it with blood pressure levels. We used a rigorous protocol to assure an empty bladder prior to initiating urine collection. We excluded subjects with a urine volume <500 mL, collection period outside of an interval of 23-25 h, and subjects with creatinine excretion that was not within the range of 14.4-33.6 mg/kg (men) and 10.8-25.2 mg/kg (women). The mean salt intake was 10.4±4.1 g/day (d), and 94% of the participants (98% of men and 90% of women) ingested more than the recommended level of 5 g/d. We found a positive association between salt and body mass index (BMI) categories, as well as with salt and blood pressure, independent of age and BMI. The difference in systolic blood pressure reached 13 mmHg between subjects consuming less than 6 g/d of salt and those ingesting more than 18 g/d. Subjects with hypertension had a higher estimated salt intake than normotensive subjects (11.4±5.0 *vs* 9.8±3.6 g/d, P<0.01), regardless of whether they were under treatment. Our data indicate the need for interventions to reduce sodium intake, as well the need for ongoing, appropriate monitoring of salt consumption in the general population.

## Introduction

High blood pressure (BP) is one of the most important risk factors for worldwide morbidity and mortality (1) and a strong surrogate for stroke (2). An increase in BP is associated with several biological, environmental, and life-style factors, including age, accumulation of body fat, physical activity, and insulin resistance. In addition, there is a large body of evidence relating high salt intake to an increase in age-dependent blood pressure and development of hypertension ([Bibr B03] [Bibr B04] [Bibr B05] 36). Studies of the association of salt intake with cardiovascular outcomes have been questioned because of methodological inconsistencies (7,8). However, Cook et al. (9) recently reported an association of sodium with cardiovascular events in a well-controlled study. The consumption of sodium is increasing worldwide, mainly secondary to the “hidden sodium” present in processed foods (10). Sodium intake and sources of dietary sodium vary among countries and different regions of specific countries. Moreover, there are different patterns of consumption among subgroups of individuals of a single population. Therefore, identifying and monitoring the distribution of sodium intake in the population as a whole prior to any public health planning and intervention should be mandatory.

Sodium consumption can be estimated using food-consumption questionnaires or urine collection. Data regarding sodium consumption in Brazil that are based on urinary collection are still scarce, mainly considering the use of the gold standard method based on 24-h urine collection (11,12).

Therefore, the main objective of this study was to determine baseline salt intake levels and their distribution in a randomized sample of the adult population of Vitoria (Brazil). This study also aimed to establish the relationship between salt consumption based on a controlled 24-h urine collection protocol and blood pressure levels.

## Material and Methods

### Population and study design

An observational and cross-sectional population-based study was carried out in the urban population of Vitória (315,000 habitants). The sample size that was necessary to estimate salt consumption with a precision of ±0.5 g/d (alpha=5% and estimated standard deviation of 4.2 g/d) was 275. An additional 20% was added to compensate for eventual losses. We obtained a final sample of 330 subjects. The sampling process was carried out in two stages. First, 20 censitary sectors were selected by lot. Second, in each sector, 20 houses with permanent residents were visited and one adult (aged 18-69 years) was invited to participate in the study. In each sector, the proportion of men and women was nearly 50%. Ages (by decades) were also planned to be equally distributed by decades. Exclusion criteria included pregnant or breastfeeding women and individuals with acute diseases, and those who were bedridden or with limited mobility (wheelchair users). After explanation of the study purpose and procedures, all participants gave written informed consent. The project was approved by the Ethics Committee of the Centro de Ciências da Saúde, Universidade Federal do Espírito Santo.

### Home visit: instructions and data collection

A home visit by research assistants was conducted to obtain sociodemographics, self-reported diseases, use of medicines, and to schedule the day of exams and urine collection. Subjects were asked about their self-identification in relation to race/ethnicity. A total of 46.3% of subjects considered themselves to be white, 10.3% were black, and 42.6% were brown or “pardo”. Two subjects reported an indigenous ancestry. Formal education was determined by years in school and the economic status was determined according to the subject’s income per month. All of the participants were asked to maintain their usual food intake habits and work routine during the day of urine collection, as well as to avoid exercising to reduce sweating. Complete oral and written instructions regarding 24-h urine collection, handling, and storage in the refrigerator were provided, as well as a warning about the common errors made during the collection (missed void, unexpected voiding commonly occurring during a shower and/or at bowel movements, and/or incorrect registering in the diary of the timing when the beginning and end of urine collection occurred). The instructions emphasized that, on the day scheduled for urine collection, subjects needed to note in a diary the exact time of first voiding in the morning, and needed to attend the University Hospital under fasting conditions to collect blood and perform clinical exams.

A venous blood sample was collected in the morning under fasting conditions (10-14 h). After voiding in a collection flask, all of the participants were subjected to clinical exams in the morning. Body weight (Toledo Scale, Brazil; 50 g precision), height (Seca Stadiometer [0.5 cm precision]; Seca GmBH & Co., Germany), and waist circumference (WC, inextensible plastic tape, 1 cm precision) were obtained by trained technicians using standard methods (13). Body mass index (BMI) was calculated as the ratio between weight and the squared height (kg/m^2^). Free fat mass (FFM) and fat mass were determined by electrical bioimpedance (InBody230, InBody, South Korea). BP and heart rate were measured in triplicate in the left arm in the seated position after a 5-min rest period, using an automated oscillometric device (Omron 765CP, Omron, USA). The mean of the two last readings was considered as the resting BP and heart rate. Participants were considered as hypertensive with a BP ≥140/90 mmHg or when using antihypertensive drugs. A conventional 12-lead electrocardiogram was also obtained (Burdick Atria 6100, USA). Samples of venous blood and 24-h urine were sent to a central laboratory to determine blood glucose, creatinine, cholesterol, lipoprotein, and triglyceride levels, and urinary sodium, potassium, and creatinine levels by using validated commercial kits. Diabetes was defined as fasting glucose >125 g/dL or use of hypoglycemic drugs. Smoking was considered present if the subject declared current use of tobacco cigarettes or a pipe, or cessation of smoking in the last year.

### Urine collection and validation procedures

Upon arriving at the Hospital Clinic, the time of the last void in the toilet in the morning was confirmed to establish the beginning of the 24-h urine collection period. Participants then received two plastic bottles (2.5 L each) to collect all of the urine until the next morning. The exact time of this last collection was written in the diary.

Completeness of the urine collection was investigated when urine bottles were taken at the participant's home by research assistants the next morning. A 24-h urine collection was considered inadequate when one or more voidings were lost, total volume was less than 500 mL, and when the collection period was outside of the interval of 23-25 h. Fourteen participants were excluded, including eight because of difficulties in following recommendations, four because the urinary volume was <500 mL, and two because the collection time was not within 23-25 h. Urinary flow was determined by dividing the total urine volume by the collection time according to the annotation in the diary. A total of 316 samples of urine were sent to a central laboratory to determine levels of Na, K, and creatinine. Total excretion of these substances was adjusted to 24 h. After urine samples were analyzed, another validation criterion based on 24-h creatinine excretion corrected to body weight was used. Only urine containing creatinine excretion in the range of 14.4-33.6 mg/kg (men) and 10.8-25.2 mg/kg (women) was accepted (11). Therefore based on this criterion, 44 participants were excluded. The final sample comprised 272 participants (82.4% of the sample). Salt consumption in these individuals was estimated considering all the urinary sodium ingested as NaCl.

### Statistical analysis

Statistical analysis was carried out using SPSS 21.0 (USA). Data are reported as means±SD and medians for continuous variables, and proportions and percentages for categorical values. The goodness of fit to a normal distribution was evaluated using the Kolmogorov-Smirnov test. Associations between gender and the studied variables were assessed by unpaired Student's *t*-test for quantitative, normally distributed variables and the Mann-Whitney test was used for non-normally distributed variables. Proportions between groups were compared by the χ^2^ test. One-way ANOVA was used when more than two means were compared, followed by Tukey's *post hoc* test. The associations of salt intake with systolic and diastolic BP and estimated FFM mass were determined by Pearson's linear correlation coefficient. A two-way ANOVA followed by the Bonferroni post hoc test was used to compare means among salt intake categories with systolic and diastolic BP, with adjustment for confounding factors (age and BMI). Statistical significance was set at P<0.05.

## Results

The final sample (n=272) comprised 129 men and 143 women, with a mean age of 44±14 years, with no differences between genders (P=0.41). Most of the variables that were measured in blood were similar between men and women, except for plasma creatinine levels, which were higher in men than in women (0.9±0.2 *vs* 0.7±0.2 mg/dL; P<0.01), and HDL-cholesterol levels, which were higher in women than in men (43±10 *vs* 51±12 mg/dL; P<0.01). The prevalence of hypertension was 31%, diabetes was 7%, smoking was 13%, and obesity (BMI ≥30 kg/m^2^) was 23%, with no significant difference between genders. Among hypertensive subjects (n=85, 31%), 27 (31.8%) were not taking antihypertensive drugs, 32 (37.6%) were on antihypertensive non-diuretic drugs, seven (8.2%) were taking only diuretics, and 19 (22.4%) were using both diuretic and non-diuretic antihypertensive drugs. Controlled BP was found in 69% of hypertensive subjects under drug treatment. Diabetes was found in 19 (7%) participants, with three subjects using both insulin and oral hypoglycemic drugs, and 16 were taking only oral drugs. Fasting glucose levels higher than 125 mg/dL were found in nine subjects.

Many of the participants had a high education level, regardless of gender. Distribution of the group by self-reported ethnicity was similar to that described in the 2010 Census for Espírito Santo State, with a predominance of white and brown groups in both genders.

**Figure 1:**
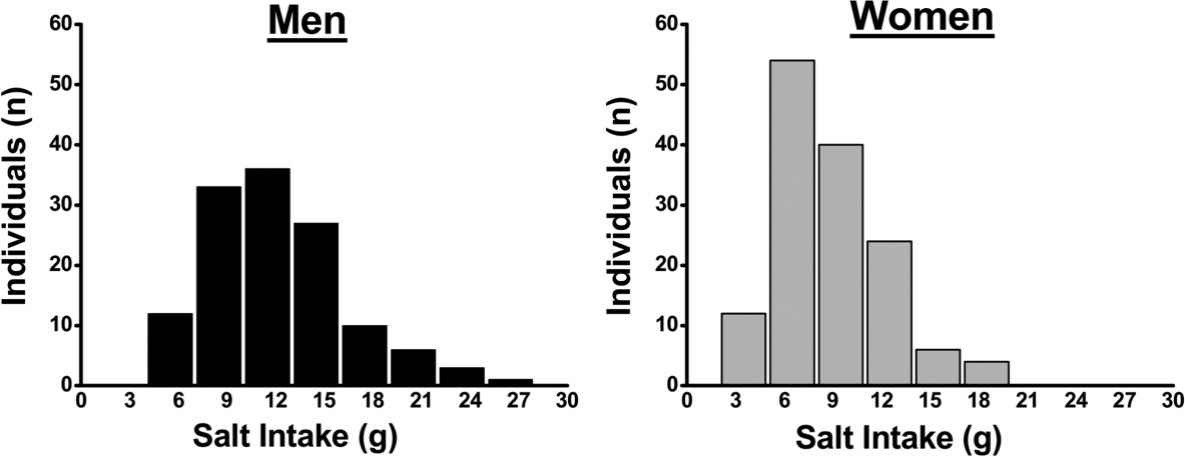
Distribution of salt intake by gender.

Table 1 shows the characteristics of urine that was collected in the 24-h period. Urine volume was similar in both genders. Twenty-four-hour creatinine excretion was higher (P<0.05) in men than in women. However, when this parameter was corrected for FFM, the difference between genders disappeared. To estimate daily salt intake, we assumed that all of the sodium that was eliminated in the urine (4060±1631 mg) came from the diet and was ingested as NaCl. We also assumed that 77% of dietary potassium was excreted in the urine, based on previous reports where urinary potassium excretion was measured in volunteers with controlled potassium ingestion (14). Based on these assumptions, the estimated mean daily salt intake was 10.3±4.1 g, and the mean value was 35% higher in men than in women (11.9 *v*s 8.8 g/d; P<0.05). The estimated daily potassium intake in the overall sample was 2.9±1.2 g/d. Potassium intake was higher in men than in women (3.3±1.4 *vs* 2.6±0.9 g/d; P<0.05). The distribution of salt intake in the studied sample is shown in Figure 1. A total of 85% of the participants (95% of men and 76% of women) showed a salt intake greater than 6 g/d. If the actual recommendation of less than 5 g/d of salt is considered, then only 6.3% (2 men and 15 women) of the sample would be considered as adherent to the actual recommendations for salt intake. In contrast, potassium intake was low in almost all individuals. A total of 94% of the subjects (91% of men and 97% of women) showed an estimated potassium intake less than 4.7 g/day (d), which is the daily allowance recommended for this mineral (15).

**Table:**
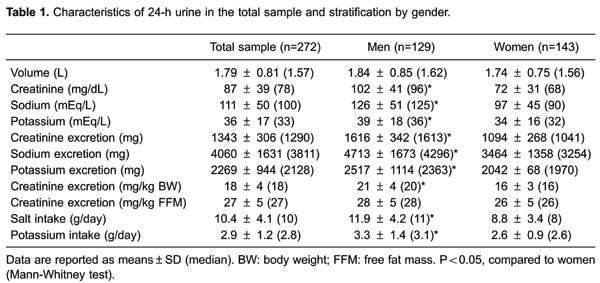

Table 2 shows estimated salt intake as a function of sociodemographic variables that were included in the present study. Salt intake was similar among the age categories, formal education levels and income levels. However, a tendency to higher ingestion was observed in black individuals with a daily salt consumption nearly 10% higher than whites. Body weight showed a clear effect on salt intake. Estimated salt intake was higher in obese subjects (11.7±4.5 g/d) compared with overweight subjects (10.7±4.2 g/d) and those with a normal BMI (9.2±3.2 g/d). These differences remained significant even after adjusting for age and systolic BP (11.7±3.0, 10.5±3.0, and 9.4±3.0 g/d, respectively; P<0.05). We did not find any difference in salt intake between subjects with and those without diabetes (9.8±3.9 *vs* 10.4±4.2 g; P=0.60). However, salt consumption in hypertensive subjects was significantly higher than that in normotensive subjects (11.4±5.0 *vs* 9.8±3.6 g/d; P<0.05). This difference persisted even after adjusting for salt consumption by age and BMI (11.3±4.1 *vs* 9.9±3.3 g/d; P*=*0.02). Inclusion of diuretics in antihypertensive treatment did not appear to affect sodium balance. This is because hypertensive individuals without treatment showed similar 24-h sodium excretion to those under regular treatment with diuretics (4.76±0.37 *vs* 4.83±0.38 g/24 h; P>0.05).

**Table:**
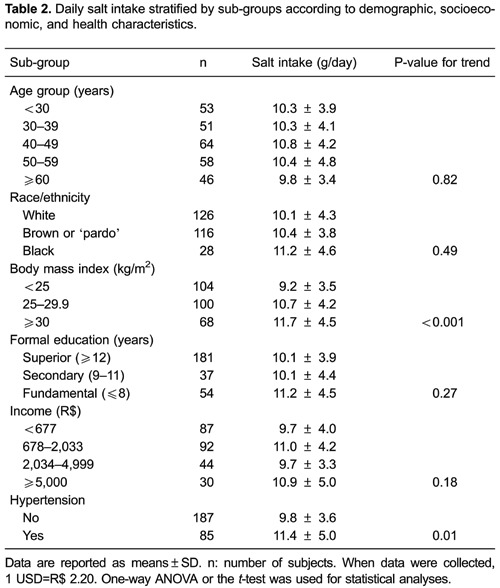

**Figure 2:**
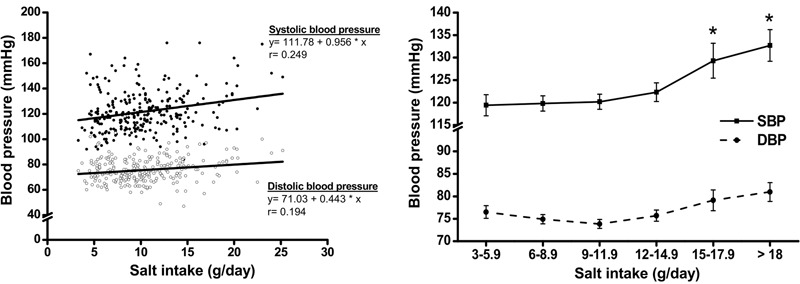
Association of salt intake with blood pressure. The left panel shows linear regression analysis between an increase in systolic and diastolic blood pressure as a function of salt intake. The right panel shows blood pressure adjusted for age and body mass index as a function of salt intake. Adjusted systolic and diastolic blood pressures were more sensitive to salt intake over 9 g/d. Data are reported as means±SE. *P<0.01 *vs* <9 g/d of salt consumption (two-way ANOVA and *post hoc* Bonferroni test).

The relationship between estimated salt consumption and BP is shown in Figure 2. Two analytical procedures were used to examine this relationship. Initially, we tested the traditional linear model and then BP was analyzed by categories of salt intake. Beta coefficients of the linear regressions showed a systolic BP increase of 0.95 mmHg (95% confidence interval [CI]=0.50-1.40 mmHg) and a diastolic BP increase of 0.44 mmHg (95% CI=0.17-0.71 mmHg) for each increment of 1 g/d of salt intake. However, the effect of sodium intake on BP may also depend on confounding variables, such as BMI (Table 2). Systolic and diastolic BP, adjusted for age and BMI, is shown as a function of salt intake categories in Figure 2. We found that the positive association between salt and systolic BP was more striking when salt intake was greater than 9 g/d. The difference in systolic BP reached 13 mmHg between subjects who consumed less than 6 g of salt and those who ingested more than 18 g of salt/d.

## Discussion

There are many studies indicating the important role of salt consumption on BP levels (6,16,17) and the high prevalence of hypertension in the Brazilian population (18). However, data on salt consumption in the general population based on urine collection are still scarce. Our study showed that the mean daily salt consumption was greater than 10 g/d, and less than 10% of the population had a sodium consumption within the actual recommendations. In contrast, potassium consumption was relatively low, suggesting an imbalance in the consumption of these minerals in the general population. Therefore, our data support the view that a great effort is necessary for improving the quality of diet that is consumed by the general population in Brazil, particularly those exhibiting overweight/obesity and hypertension. Obesity may be an important confounding factor when the relationship between salt consumption and BP is examined because obese people tend to show a higher sodium intake, even after adjustment for body weight.

The Brazilian Ministry of Health has proposed national policies to reduce sodium intake according to the National Health Plan 2012-2015 and the Strategic Action Plan to Combat Chronic Non-Communicable Diseases in Brazil 2011-2022 (19). These policies are based on data from Brazil's Family Budget Survey (POF, 2002-2003 and 2008-2009), which used food consumption records to estimate salt consumption (20,21). There are currently no national representative data using the gold standard of 24-h urine collection to measure sodium consumption. The diversity of consumption patterns that were detected in our study among people of different ancestries, education levels, and health indications (obesity and hypertension) suggests the need for a nationwide study in this area. Studies on this issue were restricted to the city of Vitória. In one study, an overnight (12 h) sample was collected in a large (n=1661) representative sample of the general adult population (22). In another study, a smaller convenience sample was used to compare urinary characteristics of the urine that was produced in the day and night (23). In both of these studies, the estimated salt intake was approximately 12 g/d. To date, the present study is the first report to describe patterns of salt consumption in a random sample of an adult Brazilian population by using the gold standard method of 24-h urine collection. We also established parameters of potassium consumption and creatinine excretion, which can be used as references for this population in future studies.

Estimates of sodium and potassium consumption based on 24-h urinary excretion depend on rigid control of the time of urine collection, which is the main source of error in studies. Therefore, we used rigorous procedures to validate this process. We initiated the 24-h period during a clinic visit to ensure that the beginning of the urine collection occurred with an empty bladder. Moreover, we excluded 24-h collection with a small volume (<500 mL) and collection periods that were not within 23-25 h. The mean and median of the collection period were close to 24 h. Moreover, we adjusted all excretions to 1440 min (24 h). The most important exclusion criterion was weight-adjusted creatinine excretion. Urine collection that occurred outside of the previously established time interval (11) was likely to have collecting errors, such as urine lost and not informed to research assistants. Importantly, the well-known difference in creatinine excretion between genders disappeared when FFM was used instead of body weight, because creatinine production is highly correlated with muscle mass (12). Additionally, appropriate collection of 24-h urine was supported by our finding of a strong correlation coefficient (*r*=0.80; P<0.001) between FFM measured by tetrapolar bioimpedance and that estimated by formula [FFM (kg)=0.02908×creatinine (mg)+7.38] using 24-h creatinine measured in urine (12). Therefore, our estimates of daily excretion of sodium and potassium were based on a rigid control process. We believe that this is the best evidence of daily consumption of sodium and potassium in the adult Brazilian population. Our sample was robust for calculating the population mean of salt consumption with a high precision (0.5 g of tolerable error). This precision cannot be translated into an individual estimate because there is substantial variation in salt in the diet from one day to another, with reflections in 24-h urinary excretion. However, our data showed good estimates for the whole population, as well as for subgroups classified by gender, age, income, ethnicity, and the presence of hypertension.

In the current study, even though salt intake did not vary regarding age, education, and income level, a tendency to higher salt intake was observed in black individuals. It is important to emphasize that our study does not have enough statistical power for sub-group analysis. However, black individuals had a 3 kg/m^2^ higher BMI compared with whites (P=0.007), and this could have been a confounding factor. Indeed, overweight and obese subjects ingested significantly higher amounts of salt than normal weight subjects, even after adjustments for age and BP. Taking BMI as a proxy for caloric intake and the significant linear correlation of BMI and salt consumption (*r*=0.36 and *r*=0.27; P<0.001 for men and women, respectively), we can assume that overweight and obese subjects appeared to consume an increased quantity of calories. These increased calories were probably the result of an increased amount of food in general. Alternatively, we speculate that obese subjects showed a preference for a diet with higher salt density. However, this issue cannot be addressed with our data because we did not use food intake records to investigate this possibility.

An important finding of our study was that more than 90% of the participants consumed more than the maximum recommended value of 5.0 g of salt/d (15). The mean intake of 10.4±4.1 g/d is similar to studies in other populations (24,25). This value was smaller than previously detected by our group in the same population of Vitória (22,23) as well as in the general Brazilian population (26). However, it is early to deduce that a decrease of salt consumption is really occurring because different methodologies to evaluate salt consumption in the present and in previous studies were used. Similar to most previous reports (10,27,28), our study also showed a higher absolute salt intake in men (11.9±4.2 g) compared with women (8.8±3.4 g), probably as a result of diverse food habits and/or increased food intake. There has been a decrease in the annual household purchase of salt from 2.98 to 2.47 kg per capita (29), and most of the dietary sodium in the Brazilian population (76.2%) comes from salt and salt-based condiments, whereas only 15.8% comes from processed foods (20), which is different to the situation in developed countries (30). However, these data may be outdated, because currently, there is a clear trend in Brazilian society toward an increased consumption of industrialized foods.

As previously reported in other studies (16,), we found a significant positive association of salt intake and BP. This association was more striking with ingestion of more than 9 g/d of salt. Additionally, differences in systolic BP reached 13 mmHg between subjects who consumed less than 6 g/d of salt and those who ingested more than 18 g/d. We also found a difference of 8 mmHg systolic BP between the highest Na/K ratio quintile (surrogate of an unhealthy diet) and the lowest quintile (surrogate of a healthy diet, data not shown). We recently showed that when people have an increased intake of potassium, a high intake of sodium is not associated with a high BP (34). In the current study, we also found that hypertensive individuals, regardless of whether they were under drug treatment, ingested significantly more sodium than normotensive individuals, even after correction for age and BMI. There is a large body of evidence showing that increased BP is the major risk factor for cardiovascular disease and the second leading modifiable cause of death (35). Hypertension was present in 30% of our sample where 74.2% of men and 43.6% of women ingested more than 9 g/d of salt. Therefore, there is an urgent need for a population-wide reduction in sodium consumption, mainly among hypertensive individuals. Successful action requires involvement of society as a whole, involving a partnership among individuals, healthcare providers, professional organizations, public health agencies, governments, and industry (36).

Our study has some strengths and limitations. The strengths of our study are that the population sample was selected at random and there was rigorous control of 24-h urine. A possible study limitation is the lack of data related to physical activity and alcohol intake. In addition, measurements of sodium based on a single 24-h urine collection fail to capture the day-to-day variability. This could have hampered the within-person monitoring of salt consumption. Moreover, the sample size was not sufficient for more robust subgroup analysis. Therefore, conclusions regarding these analyses should be considered as preliminary.

In summary, for the first time, our study identified the average salt consumption in Brazil using the gold standard method for this purpose. Only a minority of individuals followed the recommendation for sodium intake. Control of salt intake is practically absent in subjects with hypertension, irrespective of the use of antihypertensive therapy. Considering the regression analysis (Figure 2), our data suggest that systolic and diastolic BP will decrease 1 mmHg and 0.5 mmHg, respectively, for each 1 g/d reduction of salt intake in the adult population.
